# Glomerulonephritis triggered by chronical aortic graft infection in a male with Loeys–Dietz syndrome: A case report

**DOI:** 10.1097/MD.0000000000015496

**Published:** 2019-05-03

**Authors:** Xu-jie Zhou, Li-jun Liu, Pei-xin He, Fu-de Zhou

**Affiliations:** a Renal Division, Peking University First Hospital, Peking University Institute of Nephrology, Key Laboratory of Renal Disease, Ministry of Health of China; b Key Laboratory of Chronic Kidney Disease Prevention and Treatment (Peking University), Ministry of Education; c Department of Cardiology, Peking University First Hospital; Beijing, China.

**Keywords:** aortic graft, corticosteroid therapy, infection-related glomerulonephritis, Loeys–Dietz syndrome

## Abstract

**Rationale:**

Glomerulonephritis triggered by a chronically infected graft is increasingly identified because of widely used implanted device. Removal of the aortic graft and sustained antibiotic therapy is the usual approach to maximize the chance of renal recovery, but as this case shows graft removal is not always possible.

**Patient concerns:**

A 35-year-old man with intractable and recurrent fever had acute renal failure in sustained antibiotic therapy.

**Diagnoses:**

Renal biopsy suggested crescentic glomerulonephritis. ^18^fluorodeoxyglucose/positron emission tomography–computed tomography showed increased metabolic activity at the site of aortic graft, reminding that chronic infection of an implanted graft can lead to severe glomerulonephritis. *TGFBR2* c.1133G>T mutation was observed in mutation analysis, which was reported to be associated with Loeys–Dietz syndrome.

**Interventions:**

Although infection was properly controlled with appropriate antimicrobial treatment, his renal dysfunction did not improve. A short-term inclusion of low-dose corticosteroid significantly benefit without introducing harm.

**Outcomes:**

He partly recovered from renal injury.

**Lessons:**

In patients with glomerulonephritis triggered by a long-duration infection, low-dose corticosteroid therapy may be considered when renal dysfunction secondary to nephritis does not improve after appropriate antimicrobial treatment.

## 1 Introduction

In the past, bacterial infection-related glomerulonephritis (IRGN) was mostly postinfectious glomerulonephritis observed in children following streptococcal upper respiratory tract or skin infections.^[1,2]^ However, there has been considerable shift in epidemiology, bacteriology, and outcome over the past 3 to 4 decades. Increasing prevalence was observed in adults with infection-related glomerulonephritis. Disease course and outcome were significantly unfavorable compared to that of children.^[1–4]^ Those patients in elderly age or diabetics may not recover renal function. Infection-related glomerulonephritis, always an immune-complex-mediated disease characterized by necrotizing and crescentic glomerular lesions, deserves appropriate antimicrobial treatment.^[1,5–8]^ Because of increased risk of aggravating the infection responsible for the disease, corticosteroid therapy is generally not recommended. However, proper use should be considered in patients with glomerulonephritis, triggered by a long-duration infection, whose secondary renal dysfunction does not improve after appropriate antimicrobial treatment.^[2,5,6,8]^

## 2 Case report

A 35-year-old man admitted to our hospital due to acute kidney disease (serum creatinine increased to 229 μmol/L from 132 μmol/L in <3 months previously). Regarding the patient's medical history, he underwent a surgical replacement of the whole aortic arch and stent trunk 6 years ago, and was diagnosed to have hypertension and diabetes since then. He had intermittent fever (38–39°C) in the last year. Laboratory investigations revealed the following alterations, including raised C-reactive protein, raised procalcitonin and gram-negative bacteremia of *Pseudomonas aeruginosa*. He received 3 cycles of long-term (7–9 weeks per cycle) intravenous antibiotics (including 1st cycle of piperacillin/sulbactam for 2 months, 2nd cycle of meropenem for 7 weeks, 3rd cycle including meropenem 2 weeks, piperacillin/sulbactam + levofloxacin 4 weeks, ceftazidime 3 weeks) in local hospital along the past year. Although his symptoms resolved rapidly with intravenous infusion of antibiotics and blood cultures repeatedly showed negative during therapeutic regimen; he had recurrent bacteremia of the same strain isolated within a couple of days after antibiotics withdrawal. At the end of the 3rd cycle of intravenous antibiotics, it was observed that he had decreased renal function, microhematuria and proteinuria of nephrotic range. Urinalysis showed protein 3+ and urinary occult blood 3+ (200–250/high power field) with dysmorphic erythrocytes; urinary protein excretion was 6.6 g/d (previous urinalyses had been unremarkable). Further workup showed increased immunoglobin (Ig) G 25.10 g/L (7.23–17.85) but normal IgA, IgM, complement C3, C4. And antistreptolysin O, rheumatoid factor, circulatory immune complex, antineutrophil cytoplasmic antibodies, antiglomerular basement membrane antibodies, antinuclear antibody, antiextractable nuclear antigens antibodies, antiphospholipase A2 receptor antibodies, cryoglobulin, and immunofixation electrophoresis were all negative. He presented transient gross hematuria under no obvious predisposing causes and then received renal biopsy.

**Figure 1:**
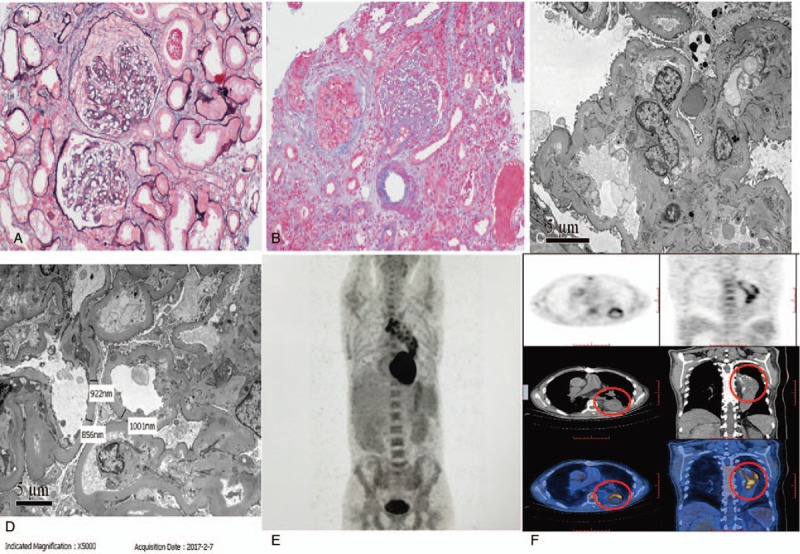
Image examination of the case. Histologic examination of a renal biopsy sample (A–D) showed 28 glomeruli, 1 was globally sclerotic, and the nonsclerotic glomerular segments had foci of increased mesangial matrix and increased mesangial cells, including 10 cellular, 2 small cellular, and 1 small cellular fibrous crescents formation; there was no duplication of basement membranes. Immunohistology showed positive mesangial staining for immunoglobin (Ig) M, albumin; whereas IgA, IgG, C1q, C3, and FRA were negative. The cortex and outer medulla had acute tubulointerstitial damage with renal tubular epithelial vacuole degeneration, multiple brush bristles off, and multifocal atrophy; interstitial matrix expansion and focal interstitial infiltrate of lymphoid and mononuclear cells, plasma cells, neutrophils, and eosinophils. And arteriole hypertrophy was observed. Congo Red reaction was negative. Electron microscopy showed mesangial regions with increased matrix and segmental subendothelial and mesangial osmiophilic deposits. These findings indicated focal mesangial proliferative glomerulonephritis with immune-complex-mediated crescentic formations as well as tuberous diabetic sclerosis. ^18^F-fluorodeoxyglucose/positron emission tomography–computed tomography (^18^F-FDG/PET-CT) showed multiple foci of increased metabolic activity at the site of aortic graft (E, F). FRA = fibrin(ogen)-related antigens.

Histologic examination of a renal biopsy sample (Fig. 1 A–D) showed 28 glomeruli, 1 was globally sclerotic, and the nonsclerotic glomerular segments had foci of increased mesangial matrix and increased mesangial cells, including 10 cellular, 2 small cellular, and 1 small cellular fibrous crescents formation; there was no duplication of basement membranes. Immunohistology showed positive mesangial staining for IgM, albumin, whereas IgA, IgG, C1q, C3, and fibrin(ogen)-related antigens were negative. The cortex and outer medulla had acute tubulointerstitial damage with renal tubular epithelial vacuole degeneration, multiple brush bristles off, and multifocal atrophy; interstitial matrix expansion and focal interstitial infiltrate of lymphoid and mononuclear cells, plasma cells, neutrophils, and eosinophils. And arteriole hypertrophy was observed. Congo red reaction was negative. Electron microscopy showed increased matrix and segmental subendothelial and mesangial osmiophilic deposits in mesangial regions. These findings indicated focal mesangial proliferative glomerulonephritis with immune-complex-mediated crescentic formations as well as tuberous diabetic sclerosis.

^18^F-fluorodeoxyglucose/positron emission tomography–computed tomography (^18^F-FDG/PET-CT) showed multiple foci of increased metabolic activity at the site of aortic graft (Fig. 1E, F). We made a diagnosis of immune-complex-mediated crescentic glomerulonephritis triggered by chronic aortic graft infection with *P aeruginosa*.^[9]^ Due to special appearance and family history of sudden cardiac death, a whole-genome sequencing was taken and a *TGFBR2* c.1133G>T mutation was observed, which was reported to be associated with Loeys–Dietz syndrome.

**Figure 2:**
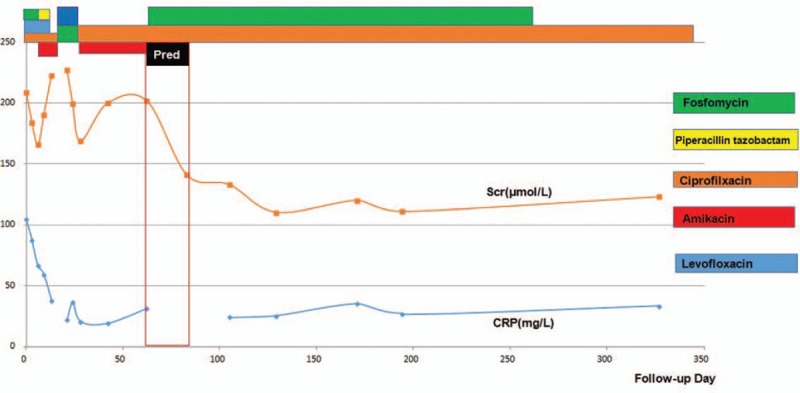
Follow-up data of the case. After 8 weeks of antibiotics of sensitive antibiotics including ciprofloxacin combined with amikacin, blood cultures remained negative, and C-reactive protein decreased to normal. But his renal function did not recover, and 2 weeks of small dose of steroid (prednisone 15 mg/d) was taken. His serum creatinine returned to normal range.

Antimicrobial susceptibility test showed multidrug resistance including all the previous antibiotics taken. The only susceptible drugs included amikacin, gentamicin, levofloxacin, and ciprofloxacin. He 1st took intravenous fosfomycin sodium combined with levofloxacin. But he cannot stand by fosfomycin because of heavy vomiting. So he received intravenous ciprofloxacin (0.4 g, q 12 hours) combined with amikacin (0.2 g, q 12 hours for 4 weeks). After 8 weeks of antibiotics of sensitive antibiotics, blood cultures remained negative and C-reactive protein decreased to normal. But his renal function did not recover, and 2 weeks of small dose of steroid (prednisone 15 mg/d) was taken. His serum creatinine returned to normal range (Fig. 2).

He continued oral antibiotics of fosfomycin sodium (1.5 g qd) and ciprofloxacin (0.2 g bid) in the following year. Up to date, the patient has been followed up for 1 year. More recent urinalysis showed negative protein and urinary blood.

## 3 Discussion

Previously, most cases of IRGN were so-called postinfectious GN occurring in children after streptococcal upper respiratory tract or skin infections. But a recent change in the epidemiology, bacteriology, disease course, and outcome of IRGN was highlighted. There has been an increased incidence of nephritis related to nonstreptococcal infection, especially in adults with differed sites of infection. Of note, the poor prognosis of sporadic adult IRGN was widely recognized.^[2,10]^ As the population ages and the number of aortic grafts placed increases, even with prophylactic antibiotics and low-infection rates, there will always be a small percentage of patients who develop aortic graft infections. Increased attention has been paid on glomerulonephritis caused by chronic low-grade infection of these “foreign bodies.”^[11–13]^ The common pathogens include gram-positive organisms, that is, methicillin-resistant *Staphylococcus aureus*, and gram-negative infections such as *P aeruginosa*, which are more prone to formations of polysaccharide biofilms.

The IRGN, always an immune-complex-mediated disease characterized by necrotizing and crescentic glomerular lesions, was more often seen in infective endocarditis.^[2,10]^ In susceptible patients, interactions among the host, pathogen, and foreign graft might trigger the development of this kind of immune complex disease. In endocarditis-associated glomerulonephritis, timely antibiotic therapy played a major role in limiting the occurrence and progression of glomerulonephritis. It was suggested that antibiotic treatment can lead to complete remission of IRGN. Nonetheless, the prognosis of IRGN depends to a large extent on the severity of the crescents and the etiology of the disease. No definitive therapeutic strategy has been confirmed for treating these patients. In graft-related infection, treatment should include both infection eradication and renal complication management. Removal of the aortic graft and sustained antibiotic therapy is the usual approach to maximize the chance of renal recovery, but as this case shows graft removal is not always possible.^[5,6]^ Such graft infection-related glomerulonephritis can lead to relentless progressive kidney failure, even with antibiotic therapy, especially if circumstances do not allow graft removal. For selected patients with aortic graft infection that cannot be removed, long-term antibiotics should be taken, starting with intravenous infusion of broad-spectrum antibiotics and followed by oral antibiotics effective against the organism permanently on the graft. However, in the current case, a short-term inclusion of low-dose corticosteroid significantly benefit without introducing harm. We suggested that corticosteroid therapy may be considered when renal dysfunction secondary to nephritis does not improve after appropriate antimicrobial treatment. But the exact dose and duration should be fully addressed and evaluated.

In this case, we further checked the genetic basis for his thoracic aortic aneurysms. This kind of disease occurs in all age groups, has a strong genetic basis, and often develops in individuals affected by hereditary connective tissue disorders. Three typical examples of hereditary connective tissue disorders are Marfan syndrome, Ehlers–Danlos syndrome, and Loeys–Dietz syndrome.^[14]^ These syndromes show some degree of phenotypical overlap of cardiovascular, skeletal, and cutaneous features. It was reported that heterozygous kinase-inactivating mutations in genes coding for either transforming growth factor-β receptor subunit (*TGFBR1* or *TGFBR2*) cause Loeys–Dietz syndrome, characterized by a highly penetrant and aggressive aneurysmal disease. No specific clinical criteria exist, as the diagnosis is confirmed by a molecular test. It highlights the importance of molecular diagnostic confirmation in phenotypic features showing overlaps.

In conclusion, in patients with glomerulonephritis triggered by a long-duration infection, low-dose corticosteroid therapy may be considered when renal dysfunction secondary to nephritis does not improve after appropriate antimicrobial treatment.

## Acknowledgment

The authors thank the patient for devotion.

## Author contributions

XJZ, PXH, and LJL were involved in the acquisition and analysis of data; wrote the manuscript; XJZ, PXH, LJL, and FDZ revised the manuscript critically for important intellectual content; FDZ supervised the whole research group and has given the final approval of the version to be published.

**Conceptualization:** Xu-jie Zhou, Pei-xin He.

**Data curation:** Xu-jie Zhou, Li-jun Liu, Pei-xin He.

**Formal analysis:** Xu-jie Zhou, Pei-xin He.

**Funding acquisition:** Xu-jie Zhou.

**Investigation:** Xu-jie Zhou, Pei-xin He.

**Methodology:** Xu-jie Zhou.

**Supervision:** Fu-de Zhou.

**Validation:** Fu-de Zhou.

**Writing – original draft:** Xu-jie Zhou, Li-jun Liu.

Xu-jie Zhou orcid: 0000-0002-7215-707X.

## References

[1] Nasr SH Radhakrishnan J D’Agati VD Bacterial infection-related glomerulonephritis in adults. Kidney Int 2013;83:792–803. 23302723 10.1038/ki.2012.407

[2] Boils CL Nasr SH Walker PD Update on endocarditis-associated glomerulonephritis. Kidney Int 2015;87:1241–9. 25607109 10.1038/ki.2014.424 PMC4455140

[3] Balasubramanian R Marks SD Post-infectious glomerulonephritis. Paediatr Int Child Health 2017;37:240–7. 28891413 10.1080/20469047.2017.1369642

[4] Khalighi MA Nguyen S Wiedeman JA Bartonella endocarditis-associated glomerulonephritis: a case report and review of the literature. Am J Kidney Dis 2014;63:1060–5. 24332768 10.1053/j.ajkd.2013.10.058

[5] Saritas T Brandenburg V Federico G Glomerulonephritis triggered by a chronically infected left ventricular assist device. Lancet 2015;386:2363–4. 26681291 10.1016/S0140-6736(15)00822-3

[6] Feehally J Glomerulonephritis from a chronically infected implanted device. Lancet 2015;386:2364. 26681292 10.1016/S0140-6736(15)00826-0

[7] Wang A Wang Y Wang G Infective endocarditis associated with acute renal failure: repeat renal biopsy and successful recovery. Exp Ther Med 2010;1:433–6. 22993558 10.3892/etm_00000067 PMC3445935

[8] Koya D Shibuya K Kikkawa R Successful recovery of infective endocarditis-induced rapidly progressive glomerulonephritis by steroid therapy combined with antibiotics: a case report. BMC Nephrol 2004;5:18. 15610562 10.1186/1471-2369-5-18 PMC544880

[9] Lyons OT Baguneid M Barwick TD Diagnosis of aortic graft infection: a case definition by the Management of Aortic Graft Infection Collaboration (MAGIC). Eur J Vasc Endovasc Surg 2016;52:758–63. 27771318 10.1016/j.ejvs.2016.09.007

[10] Lusco MA Fogo AB Najafian B AJKD atlas of renal pathology: subacute bacterial endocarditis-associated glomerulonephritis. Am J Kidney Dis 2016;68:e11–2. 27477362 10.1053/j.ajkd.2016.06.001

[11] Tihan D Aksoy M A real mycotic aneurysm-mycotic aneurysm of the abdominal aorta due to fungal infection. Ulus Cerrahi Derg 2014;30:222–4. 25931934 10.5152/UCD.2014.2703 PMC4379792

[12] Jandhyala D Farid S Mahmood M Unrecognized pre-transplant disseminated *Coxiella burnetti* infection diagnosed in a post-transplant heart-kidney recipient. Transpl Infect Dis 2018;20:e12962. 29975810 10.1111/tid.12962

[13] Nagaba Y Aoyama T Sano T Successful treatment of MRSA-associated glomerulonephritis with antibiotic therapy [in Japanese]. Nihon Jinzo Gakkai Shi 2003;45:37–41. 12680319

[14] Meester J Verstraeten A Schepers D Differences in manifestations of Marfan syndrome, Ehlers-Danlos syndrome, and Loeys-Dietz syndrome. Ann Cardiothorac Surg 2017;6:582–94. 29270370 10.21037/acs.2017.11.03 PMC5721110

